# Molecular Cytogenetic Diagnosis and Somatic Genome Variations

**DOI:** 10.2174/138920210793176010

**Published:** 2010-09

**Authors:** S.G Vorsanova, Y.B. Yurov, I.V. Soloviev, I.Y. Iourov

**Affiliations:** 1 Institute of Pediatrics and Children Surgery, Rosmedtechnologii; 2 Mental Health Research Center, Russian Academy of Medical Sciences; 3 Moscow City University of Psychology and Education, Moscow, Russia

**Keywords:** Molecular cytogenetics, somatic genome variations, molecular diagnosis, chromosome instability, genomic instability, mosaicism.

## Abstract

Human molecular cytogenetics integrates the knowledge on chromosome and genome organization at the molecular and cellular levels in health and disease. Molecular cytogenetic diagnosis is an integral part of current genomic medicine and is the standard of care in medical genetics and cytogenetics, reproductive medicine, pediatrics, neuropsychiatry and oncology. Regardless numerous advances in this field made throughout the last two decades, researchers and practitioners who apply molecular cytogenetic techniques may encounter several problems that are extremely difficult to solve. One of them is undoubtedly the occurrence of somatic genome and chromosome variations, leading to genomic and chromosomal mosaicism, which are related but not limited to technological and evaluative limitations as well as multiplicity of interpretations. More dramatically, current biomedical literature almost lacks descriptions, guidelines or solutions of these problems. The present article overviews all these problems and gathers those exclusive data acquired from studies of genome and chromosome instability that is relevant to identification and interpretations of this fairly common cause of somatic genomic variations and chromosomal mosaicism. Although the way to define pathogenic value of all the intercellular variations of the human genome is far from being completely understood, it is possible to propose recommendations on molecular cytogenetic diagnosis and management of somatic genome variations in clinical population.

## INTRODUCTION

It is hard to find a cytogeneticist who has not encountered the problem of interpreting mosaicism. It seems to be relatively simple when the majority of cells are abnormal, but is extremely difficult in low-level mosaics or in cases of detecting cryptic, complex or dynamic mosaic cell populations. With the introduction of molecular cytogenetic techniques into basic research and diagnostic practice, this has become not only a problem of practicing cytogeneticists, but a major focus of biomedicine encompassing genomics, medical genetics, neuropsychiatry, aging research and reproductive medicine [1]. Since postulating the idea that somatic genome variations (SGV) can be a source of human interindividual diversity in health and disease [2, 3], it became evident that current diagnostic research cannot leave aside this issue.

There are two main problems surrounding SGV detection during molecular cytogenetic diagnosis. The first one is technological. The majority of the researchers’ audience is unfamiliar with techniques providing for the highest resolution of SGV evaluation, even though such technologies do exist [4]. Additionally, no criteria have been delineated for definition of mosaic genome variations [1-4]. The second problem is related to interpretation of data on SGV. Succeeding in detecting mosaicism (or SGV) does not necessarily mean diagnostic success. It is usually hard to come to definite conclusion about results of scoring such a rare event as low-level mosaicism. The problem is even more complicated because there are no data on benign rates of SGV in humans. Therefore, to provide for opportunities regarding molecular cytogenetic diagnosis of SGV (mosaicism), additional large-scale studies and case-reports performed with thorough phenotype evaluations and high-resolution single-cell genome analyses are required [1-3]. 

This article overviews both problems in the light of new developments in molecular cytogenetics. Further, we have attempted to define the way it can be managed in molecular cytogenetic diagnosis of chromosome imbalances in normal and clinical population on the basis of rare data on SGV. Additionally, associations between this phenomenon and disease are described in order to simplify interpretations of mosaicism. Finally, brief troubleshooting and perspectives of SGV research in this extent are given to assist researchers in molecular diagnosis of mosaicism.

## THE ESTIMATED IMPACT OF SGV ON MOLECULAR CYTOGENETIC DIAGNOSIS

To get a view of SGV impact on molecular cytogenetic diagnosis, one has to refer to the diseases that are associated with. These are malignant [5, 6], brain [3, 7] and autoimmune diseases [8] as well as a number of hereditary and chromosomal syndromes that are associated with genome or chromosome instability [1-3, 7, 9, 10]. Numerous morbid conditions are also found to associate with SGV or chromosomal mosaicism: spontaneous fetal loss [11, 12], stillbirth, idiopathic congenital malformations or some clinical features of hereditary polygenic disorders [1-3, 11, 13]. Finally, cases of complex chromosomal and genomic rearrangements (CCRs), copy number variations (CNVs) and monogenic syndromes can exhibit SGV [14-18]. Together, it makes an appreciable contribution to human morbidity.

Molecular cytogenetic diagnosis has become an integral part of modern medical care being required for numerous fields of medicine [1-4, 19-21]. Molecular cytogenetic techniques are not equally applied for each aforementioned condition. All types of chromosomal imbalances manifesting at chromosomal or subchromsomal level (i.e. chromosomal syndromes, almost all malignant and some brain diseases, fetal losses and idiopathic congenital malformations) strongly require molecular cytogenetic techniques in contrast to genome/chromosome instability syndromes that are caused by gene mutations [2-4, 19-35]. The resolution or application need of molecular cytogenetic techniques for diagnosis is determined by DNA sequences that are involved in genomic rearrangements (for more details see [2-4, 19-21]). The molecular cytogenetic platforms that have to be mentioned in this context are FISH (fluorescence *in situ* hybridization) and CGH (comparative genomic hybridization) or, more precisely, array CGH. However, the most applicable methods for detection of SGV are based on FISH (for review see [2, 4]). Table **1** gives an overview of conditions associated with SGV that requires molecular cytogenetic diagnosis.

As one can see, morbid conditions, which are in need of molecular cytogenetics, are either those caused by subtle genomic rearrangements (detected at molecular level) or those associated with mosaicism in a proportion of cases. These are more commonly manifested as cases of chromosomal mosaicism [1]. Syndromes of chromosome instability exhibit changes in chromosomal numbers and/or structure in somatic cells, which can be defined as SGV or somatic chromosomal mosaicism [10, 36, 37]. However, being of monogenic nature, many of these diseases are usually diagnosed *via* molecular genetic techniques (sequencing, PCR). Therefore, a molecular cytogenetic technique applied in this instance has to provide simultaneously for high resolution and for single-cell analysis [4]. The next part of our brief review addresses this as well as other issues concerning technical aspects of SGV detection by molecular cytogenetic techniques.

## THE FIRST PROBLEM: TECHNICAL POTENTIAL OF MOLECULAR CYTOGENETICS FOR SGV DETECTION

Recently, a series of reviews has addressed the level of excellence in molecular cytogenetics with respect to identification of SGV [1-4, 21, 38]. It allows us to skip wide discussions about technological aspects of molecular cytogenetic analyses of human chromosomes for detection of mosaicism/SGV. Nevertheless, we would like to point some aspects relevant to the diagnosis. Firstly, we have to mention that it is really possible to achieve the resolution as high as 1% of cells affected by SGV or even lower [4, 28, 36-42]. The scoring of such a rare event as low-mosaicism is granted by approaches that include several molecular cytogenetic FISH-based techniques. These analyses possess the potential to embrace large cell populations. The latter means such assays to be more likely interphase than metaphase ones. For analyzing the majority of cell types, this is the unique possibility. Here, it is to note the existence of numerous drawbacks of the classical interphase FISH protocols: numerous genome behavioral peculiarities (replication, high-ordered chromosome organization in interphase nuclei) mimic chromosome number/structure variations (SGV), chromosomal heteromorphisms, or simply lack of visualization of whole chromosomes [4, 36, 43-47]. To get rid of these technical problems, it has been proposed to use in parallel multiprobe interphase FISH (simultaneous multitarget), digital analyses of FISH data (QFISH) and interphase multicolor banding technique for visualization of whole chromosomes in non-dividing cells [3, 12, 29, 36-44, 46, 47]. Although the approach has been exclusively applied to basic research, it has the potential to be a powerful diagnostic tool [1-4, 21].

Regardless developments in molecular cytogenetics that allow to analyze either interphase nuclei or DNA isolated from any cell type, the major part of cases requiring cytogenetic evaluation are firstly assessed through metaphase-analysis-based techniques [4, 21, 48]. This is the source of another, yet unresolved, problem — discrepancies between metaphase and interphase molecular cytogenetic analyses. To date, no clear explanation has been found to understand the basis and meaning of such differences. Eventually, one can see it looking through those few papers describing recommendations concerning detection of mosaicism in clinical cytogenetic practice or basic SGV research [2, 3, 12, 29, 36-42, 49-51]. Despite of the same ultimate aim referred to uncovering the presence of mosaicism, these guidelines differ significantly by technological requirements and the ways of the development. Taking into account the lack of major technological impediments, the problem of “mosaicism guidelines” correlation is the essential technical one.

## THE SECOND PROBLEM: WHAT DOES SGV MEAN?

As previously noted, the confirmation of mosaicism (especially, low-level mosaicism) in an individual leads to the problem of the interpretation. In other words, pathogenicity of mosaicism or SGV is always a matter of conjecture. It seems to be easier when abnormal cell lines are prevalent, but the borderline between pathogenic condition and benign variation is undetermined [3]. This is further complicated inasmuch as SGV is more commonly observed in a clinical population and is known to be associated with malignization [1-15, 25-29, 31, 32, 35-38, 42, 48]. Due to a higher frequency, the best studied in this context is aneuploidy or aneuploidization of somatic tissues [1-4]. Analyses of aneuploidy effect on transcriptional activity of the whole cellular genome yielded contradictory results, showing, however, that aneuploidization should possess a global negative effect on cellular physiology *via* transcriptional changes [52, 53]. Studies implicating clinical population, aneuploidy syndromes or chromosomally instable tissues (disease models) have shown that deleterious effects are only produced in cases of either chromosome-specific increase of aneuploidy rates or a global rise of spontaneous chromosomal mutations. Background (sporadic) aneuploidy is more likely to be a result of natural (benign) SGV without appreciable effects [2, 3, 7, 10, 27-29, 32, 36-42, 54].

Since the data on SGV were rarely confirmed to be pathogenic, it is better to consider such genomic variations rather a neutral or a susceptibility factor than a causative genetic abnormality. Unless repeatedly described in the literature, low-level mosaics or SGV cannot be defined as disease-causing mutations. It does not mean that some disease with strong genetic background do not require molecular cytogenetic diagnosis respecting the presence of SGV, but suggests more critical assessment as to constitutional genomic rearrangements. In summary, even though several lines of evidence suggest an association between low-level mosaics, SGV and an abnormal phenotype, their clinical interpretation and diagnostic value seem to require additional case-control studies.

## SOLUTIONS AND RECOMMENDATIONS

Molecular cytogenetic diagnosis of SGV means: (i) scoring of rare events; (ii) possible lack of reproducibility; (iii) different approaches may not yield similar results; (iv) detection of SGV does not imply guaranteed diagnostic success. Together, it makes an impression that the solution of all the difficulties surrounding this task is hardly possible. Notwithstanding, either separate or sequential application of numerous molecular cytogenetic techniques gives extremely high resolution for scoring single-cell rare events [1-3, 12, 36-42]. The irreproducibility is essentially produced by analysis of cells cultivated *in vitro* (scoring of insufficient number of metaphase cells or combined metaphase/interphase analyses) [4]. Therefore, application of interphase molecular cytogenetic approaches using uncultured cell populations can help to escape from such situations. This is partially confirmed by analysis of SGV in neuropsychiatric diseases [27-29, 32, 36, 37, 40-47]. These studies as well as the analysis of unaffected somatic tissues have also generated an integrated approach towards uncovering SGV affecting 1% of cells or lower [2-4, 7, 29, 36, 37, 40-47]. Thus, SGV detection meets no more technological problems than those associated with selecting benchmarks for evaluations of mosaicism in a given cell population. Although it still does not answer the question concerning the pathogenicity, the application of specified guidelines allows to make evaluations of mosaicism occurrence in large cohorts with respect to its biomedical meaning. Moreover, these data would give the possibility to determine the real amount of abnormal cells in an individual regardless any effect it can produce. Table **2** summarizes experimentally tested recommendations for detection of mosaicism (SGV).

The recommendations presented in Table **2** are unlikely to be combined for producing unified guidelines. The latter is mainly caused by different techniques applied for the development and area of application. Therefore, to solve the problem, a large-scale study of several cell populations acquired from different somatic human tissues appears to be strongly required. Nonetheless, before such data will be available, one has to follow these recommendations or to develop new ones on basis of original molecular cytogenetic investigation.

Despite of difficulties in interpreting results, there are positive associations between SGV and several morbid conditions [1-3, 7, 12, 29, 36, 37, 40-47, 54]. Since no other data is available to solve the problem of interpretation multiplicity, we found pertinent to mention all the pathologic conditions associated with SGV. 

SGV manifesting as chromosome-specific mosaicism or instability are common among spontaneous abortions in the first trimester [12]; chromosomal, microdeletion/duplication and CNV-associated syndromes [3, 7, 11, 13, 25, 45, 48, 54]; chromosome instability syndromes (ataxia-telangiectasia) [37]; supernumerary marker chromosomes [55-57]; idiopathic learning disability with/without congenital malformations [1-4, 7, 58, 59]; autism [27-29]; schizophrenia [31, 32, 42]; autoimmune diseases [8]; Alzheimer’s disease [36]. This type of SGV is also a susceptibility factor for female germline aneuploidization leading to trisomic conceptuses (trisomy of chromosome 21) [60] and a “license to live” for males with X-linked dominant diseases (i.e. Rett syndrome) who need an additional chromosome X to escape intrauterine lethality [61]. CCRs are primarily associated with reproduction problems [14, 15, 17] and more rarely with learning disability and congenital malformations [17]. SGV manifesting as increased levels of genome/chromosome instability, spontaneous chromosomal mutations or aneuploidization are observed in cancers or cancer-predisposition syndromes [2, 5, 6, 9, 10, 13]; chromosome instability syndromes (ataxia-telangiectasia) [9, 10, 36, 37]; idiopathic learning disability with/without congenital malformations [1-4, 7, 58, 59]; schizophrenia [31, 32, 42]; diseases of pathological/accelerating aging [10]. Another important diagnostic problem related to molecular cytogenetics of SGV is dynamic mosaicism. The latter is indeed relatively easy to solve: a series of high-resolution metaphase/interphase molecular cytogenetic techniques has to be used [62]. Finally, it is supposed that numerous other diseases could be associated with SGV [1-4]. 

## THE UPDATED SCHEME FOR MOLECULAR CYTOGENETIC DIAGNOSIS

**Fig. (1):**
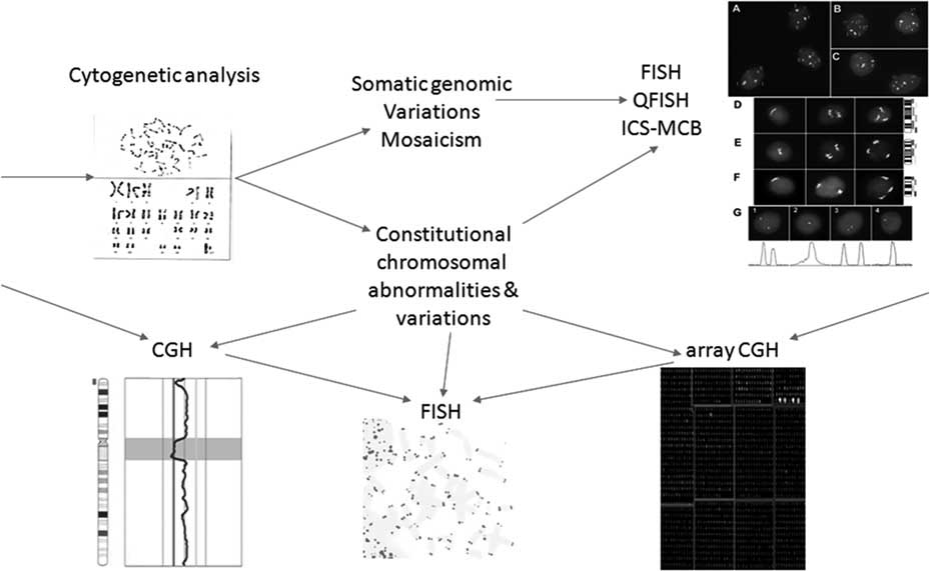
A proposed scheme of molecular cytogenetic diagnosis including detection of chromosomal mosacism or SGV manifesting at chromosomal (subchromosomal) level. Following arrows, one can get a sequence of analyses to be performed for high-resolution molecular cytogenetic diagnosis (arrows from outside of the figure indicate those techniques that provide for whole genome analysis having, thereby, potential to be applied as starting ones). The sequence, however, is not mandatory and is usually defined according to laboratory experience or case singularities. Part of the figure symbolizing “FISH QFISH ICS-MCB” is a reproduction of a figure from Yurov *et al*. [41], an openaccess article distributed under the terms of the Creative Commons Attribution License. ICS-MCB — interphase chromosome-specific multicolor banding (for details see [40, 47]).

Taking into account all the developments in the field of molecular cytogenetics as well as main characteristics of more popular FISH-, CGH- and array-CGH-based techniques [1-4, 19-21, 26, 34-47, 63, 64], we have made an attempt to propose an original diagnostic scheme that include the analyses of mosaicism and SGV. Fig. (**1**) depicts the proposed scheme of molecular cytogenetic diagnosis taking into account all the points discussed herein. It is noteworthy that previous schemes of molecular diagnosis in the available biomedical literature have never addressed the detection of SGV manifested at chromosomal or subchromosomal level (chromosomal mosaicism). Together, one can get an idea of the huge amount of “diagnostic work” that has to be performed for high-resolution diagnosis of the mosaicism. Additionally, “starting techniques” have to be those providing for whole genome screen (i.e. cytogenetic banding analyses, standard (high-resolution) CGH or array CGH). However, it is to keep in mind that CGH-based techniques are poorly applicable for low-level mosaicism and SGV detection in large cell populations [4]. FISH-based techniques (multiprobe FISH and quantitative FISH) allow targeted single-cell analysis of genomic loci in huge cell populations and only multicolor banding analysis depicts whole chromosomes in non-dividing cells (for more details see Fig. **1** and [1-4, 21, 38]). We suggest our proposal to lead to more effective molecular cytogenetic diagnosis in cases of mosaicism or SGV.

## CONCLUDING REMARKS

It is hard not to recognize that current biomedical diagnostic research and practice are more focused on constitutional genomic rearrangements that are easier to interpret and to use for disease-association studies [63, 64]. As a result, numerous human morbid conditions are postulated to require molecular cytogenetic diagnosis by array-CGH-based techniques [17, 20, 24-26, 30, 33, 54, 63, 64], which operate with total DNA isolated from a pool of cells and are inapplicable for studying the majority of mosaicism types and SGV [1-4, 7, 19, 21, 23, 27-29, 36-42, 47, 54, 57, 62]. Therefore, high-resolution whole genome screen is not the complete diagnostic solution for current molecular diagnosis. SGV require a specific set of molecular cytogenetic techniques to be diagnosed (Fig. **1**). Thus, to provide a highly technological medical care (at least, in diagnostic terms), these have to be introduced. 

The problems that surround diagnosis of SGV and mosaicism remain to be thoroughly addressed by large-scale forthcoming studies targeted at definition of causative amount of abnormal cells per analysis, local effect on cellular/tissular physiology with respect to potential effects on the whole organism, correlations between metaphase and interphase analyses in cases of molecular cytogenetic diagnosis of chromosome abnormalities. To this end, we would like to point out that current molecular cytogenetics possess tools for high-resolution detection of SGV, which have to be used for uncovering the biomedical significance of this common and, probably, most mysterious type of genomic variations.

Hopefully, further research will be able to solve this complex and widely relevant problem.

## Figures and Tables

**Table 1.** Diseases and Morbid Conditions Associated with SGV Requiring Molecular Cytogenetic Diagnosis

Disease/Morbid Condition	Platforms for Diagnosis	Necessity of Molecular Cytogenetic Diagnosis*	Key Refs
Spontaneous abortions (10-15 wks)	FISH	+++	[12]
Abnormal prenatal development (prenatal diagnosis)	FISH	+++	[11, 22-24]
	CGH**		
Chromosomal syndromes/non-specific causal abnormalities	FISH	+++	[1-4, 11, 19-21]
	CGH		
Diseases caused by CNVs	CGH	+++	[25]
	FISH		
Idiopathic congenital malformations	FISH	+++	[2, 11, 19-21]
Developmental delays	CGH		
Idiopathic learning disability	FISH	+++	[7, 11, 26]
	CGH		
Cancer	FISH	+++	[2, 4-6, 19-21]
	CGH		
Autism	FISH	++	[7, 27-30]
	CGH		
Schizophrenia	FISH	+	[31-33]
	CGH		
Autoimmune diseases	FISH	+	[8]
Monogenic syndromes	Fiber FISH	+	[34, 35]

* –the level of necessity is defined as indispensable

** –array CGH included.

++ rarely applied (+);

+++ method of choice

**Table 2.** Recommendations for Detection of Chromosomal Mosaicism and SGV

Source	Area of Application	Detection Rate	Amount of Cells to Score
Hsu *et al*., 1992 [49]	Prenatal diagnosis by cytogenetic techniques	0.5% (1 cell)*	200
Caudill *et al*., 2005 [50]	Prenatal diagnosis by cytogenetic techniques	15%	15-30
Vorsanova *et al*., 2005 [12]	Fetal aneuploidy by molecular cytogenetic techniques	<5%	300-500
Yurov *et al*., 2005 [39]; 2007 [29, 41]; 2008 [42]; Iourov *et al*., 2006 [3]; 2009 [38].	Chromosome abnormalities or SGV by molecular cytogenetic techniques	<0.1%	1000-10000
Iourov *et al*., 2009 [36, 37]	Chromosome instability or SGV by molecular cytogenetic techniques	0.1-1%	1000-10000
Iourov *et al*., 2006 [40]; 2009 [38]	Chromosome abnormalities or SGV by molecular cytogenetic techniques	<0.5% (1 cell)*	>100
Wiktor *et al*., 2009 [51]	Sex chromosome aneuploidy by cytogenetic techniques	>3% (1 cell)	20-30

* – pseudomosaicism.
